# Can enzyme proximity accelerate cascade reactions?

**DOI:** 10.1038/s41598-018-37034-3

**Published:** 2019-01-24

**Authors:** Andrij Kuzmak, Sheiliza Carmali, Eric von Lieres, Alan J. Russell, Svyatoslav Kondrat

**Affiliations:** 1 Department for Theoretical Physics, I. Franko National University of Lviv, Lviv, Ukraine; 2 Department of Chemistry, Aarhus University, 8000 Aarhus C, Denmark; 3 Center for Polymer-Based Protein Engineering, Carnegie Mellon University, 5000 Forbes Avenue, Pittsburgh, PA 15213 USA; 4 Forschungszentrum Jülich, IBG-1: Biotechnology, 52425 Jülich, Germany; 5 Department of Chemical Engineering, Carnegie Mellon University, 4400 Fifth Avenue, Pittsburgh PA, 15213 USA; 6 Department of Complex Systems, Institute of Physical Chemistry, Warsaw, Poland

## Abstract

The last decade has seen an exponential expansion of interest in conjugating multiple enzymes of cascades in close proximity to each other, with the overarching goal being to accelerate the overall reaction rate. However, some evidence has emerged that there is no effect of proximity channeling on the reaction velocity of the popular GOx-HRP cascade, particularly in the presence of a competing enzyme (catalase). Herein, we rationalize these experimental results quantitatively. We show that, in general, proximity channeling can enhance reaction velocity in the presence of competing enzymes, but in steady state a significant enhancement can only be achieved for diffusion-limited reactions or at high concentrations of competing enzymes. We provide simple equations to estimate the effect of channeling *quantitatively* and demonstrate that proximity can have a more pronounced effect under crowding conditions *in vivo*, particularly that crowding can enhance the overall rates of channeled cascade reactions.

## Introduction

Enzyme-catalyzed reactions are probably the most ubiquitous and elegant reactions on Earth. Typically an enzyme-catalyzed reaction does not occur alone, but instead is a part of a natural metabolic pathway or synthetic cascade. A particular role in enzymatic cascades is played by metabolite or substrate channeling, in which the product of one reaction is directly passed to the active site of the next enzyme in a cascade, either via a physical tunnel in an enzyme-enzyme complex^1–4^, along an ‘electrostatic highway’^3–6^, or through proximity of two (or more) enzymes^7–10^.

Substrate channeling *in vivo* has also been a subject of yet to be resolved debates^11–15^. Despite work showing the existence of enzyme-enzyme complexes inside living cells^16–21^, a consensus has not emerged as to whether, and to what extent, channeling occurs in cells and how it influences reaction velocities. In particular, Poshyvailo *et* *al*.^22^ have argued that direct channeling may slow down the reaction velocity and increase the metabolite pool size, while its main benefit is likely to protect metabolites from degradation or competing side reactions.

In biotechnology, on the other hand, a significant effort has been channeled into developing novel methods to bring and keep enzymes together, hoping to accelerate the reactions by decreasing the diffusion path between the enzymes^7–9,21,23–33^. Fu *et al*.^26^ have assembled glucose oxidase (GOx) and horseradish peroxidase (HRP) enzymes on a DNA origami and reported a 15-fold increase of the overall reaction rate. However, in a recent paper, Zhang *et al*.^34^ suggested that the enhanced velocity was due to an increased pH at the origami tile, while the reaction velocity was essentially insensitive to the enzyme proximity under conditions close to the steady state. The same conclusion was also reached in the presence of a competing enzyme (catalase), contrary to expectations.

**Figure 1:**
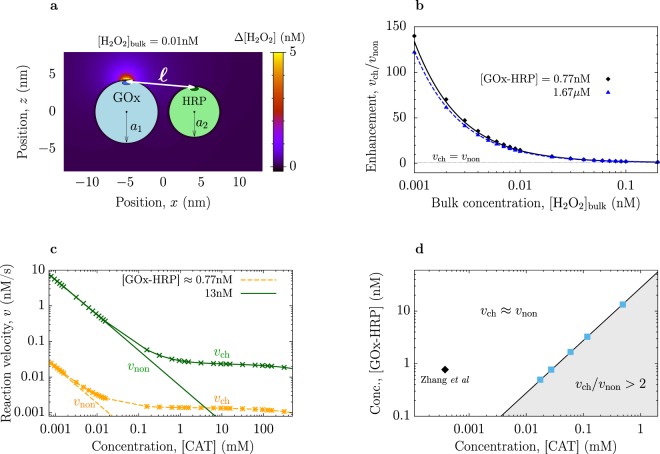
Effect of proximity channeling on GOx-HRP cascade. (**a**) A model of a GOx-HRP complex and the distribution of hydrogen peroxide (H_2_O_2_, intermediate of the GOx-HRP cascade) shown as a deviation from the bulk concentration, Δ[H_2_O_2_] = [H_2_O_2_] − [H_2_O_2_]_bulk_. (**b**) Enhancement *v*_ch_/*v*_non_ due to proximity channeling as a function of the bulk concentration of hydrogen peroxide. Symbols show the results of the full numerical calculations and the lines have been obtained using equation (1). (**c**) Reaction velocities of the channeled (symbols) and non-channeled (lines) reactions as functions of concentration of catalase (enzyme competing with HRP for hydrogen peroxide). (**d**) Channeling diagram showing the region where the enzyme proximity can at least double the reaction velocity. The diagram is drawn in the plane of the concentration of enzyme complexes and the concentration of catalase. We have assumed that [GOx-HRP] is the same as [GOx] = [HRP] in the non-channeled system. Squares show the results of the full numerical calculations and the line has been obtained using equation (3). The diamond shows the system with the maximal concentration of catalase studied experimentally by Zhang *et al*.^34^.

Herein, we analyse proximity channeling in detail and further clarify if and when enzyme proximity would be beneficial to reaction velocity. To achieve this goal we use a theoretical model, in which the concentration of intermediates was obtained in the presence of two enzymes of a cascade (Fig. 1a). We then solved the appropriate diffusion problem and the reaction rates were computed and compared with the corresponding rates of a non-channeled system (see Methods).

Before discussing the results of those calculations, however, it is vital to stress, again^27,35,36^, that, in steady state, enzyme proximity *cannot* accelerate the reaction velocity in the absence of competing enzymes or degrading intermediates. Indeed, the conservation law dictates that for a tandem reaction (a cascade of two enzyme-catalyzed reactions), the rate of production of intermediates and the rate of their conversion into the final product must be equal in steady state (otherwise the concentration of intermediates would change over time). This means that the reaction velocity is influenced only by the production rate of the first enzyme, which is the same in the channeled and non-channeled system (see equations (6) and (11) in Methods). The conservation-law argument is mathematically rigorous and independent of the position of enzymes, diffusion rates and other parameters. Thus, we believe that enzyme proximity *cannot* accelerate reactions at low enzyme concentrations or for ‘perfect enzymes’, as suggested in a recent review^15^. In steady state, an intermediate does not have to diffuse *directly* between the enzymes for the reaction to occur, since it is already available in the bulk solution; in other words, the diffusion path of an intermediate is determined by the concentration of the second enzyme and by the concentration of intermediates, which are ultimately non-zero in steady state (see equation (10) with *k*_deg_ = 0 in Methods).

We decided to consider the effect of proximity before steady-state is achieved. In Fig. 1a we plot the distribution of intermediates (hydrogen peroxide) for the GOx-HRP system of Zhang *et al*.^34^ (Supplementry Note S1). For this relatively low non-steady state bulk concentration of the hydrogen peroxide, [H_2_O_2_]_bulk_ = 0.01 nM, the enzyme proximity had a dominant contribution on the reaction velocity. Indeed, for the non-channeled system the reaction velocity was *v*_non_ = *k*_HRP_[HRP][H_2_O_2_]_bulk_ = 0.1 pM/s, and *v*_ch_ ≈ 1.5 pM/s for the channeled system, giving the enhancement *v*_ch_/*v*_non_ ≈ 15 (albeit at extremely low reaction rates). At higher bulk concentrations, however, the difference Δ[H_2_O_2_] = [H_2_O_2_]_at−HRP_ − [H_2_O_2_]_bulk_ became small, as compared to [H_2_O_2_]_bulk_, rendering proximity channeling useless. Enzyme proximity accelerated the GOx-HRP cascade at very low H_2_O_2_ concentrations, with saturation occurring at a concentration as low as 0.1 nM (Fig. 1b). Assuming [H_2_O_2_] = 0 at time *t* = 0, we estimated that the GOx-HRP cascade would benefit from proximity channeling merely within the first *τ*_1_ ≈ 87 ms (see equation (9) in Methods); for other systems *τ*_1_ could be larger^15,36^. However, we shall show that enzyme proximity can accelerate cascade reactions also in steady state, but *only* in the presence of competing enzymes or if intermediates degrade. A similar conclusion has been reached by Idan and Hess^36^, who related reaction acceleration to the ratio between the life-time of intermediates and the characteristic time of temporal boost (analogous to our *τ*_1_). Here, we provide an alternative clarification and derive simple expressions to estimate rate enhancement quantitatively.

In order to achieve a deeper insight into the proximity-induced enhancement, we solved analytically the diffusion problem for intermediates and the first enzyme of a cascade; then, we estimated the rate enhancement by looking at the excess concentration of intermediates at the location of the active site of the second enzyme. Limiting our considerations to the first term in an infinite series expansion of the exact solution, we have arrived at a simple approximate equation (Supplementry Note S4)

1 $${v}_{{\rm{c}}{\rm{h}}}/{v}_{{\rm{n}}{\rm{o}}{\rm{n}}}\approx 1+\frac{{k}_{{{\rm{E}}}_{1}}}{{k}_{\ell D}{[{\rm{I}}]}_{{\rm{b}}{\rm{u}}{\rm{l}}{\rm{k}}}}(1-\ell /b),$$

where [I]_bulk_ is the bulk concentration of intermediates (taken the same in the channeled and non-channeled systems), $\ell$ is the distance between the active sites of the two enzymes in a complex (Fig. 1a) and *b* = (3/4*π*[E_1_])^−1/3^ roughly corresponds to the average distance between the enzymes in the non-channeled system (here [E_1_] is the enzyme concentration). The reaction constant (in units of s^−1^) is ${k}_{{{\rm{E}}}_{1}}=[{\rm{S}}]{k}_{{\rm{cat}}}^{(1)}/{K}_{M}^{\mathrm{(1)}}$, assuming $[{\rm{S}}]\ll {K}_{M}^{\mathrm{(1)}}$, where [S] is the concentration of the first enzyme’s substrate (glucose in the case of GOx) and *k*^(1)^ and ${K}_{M}^{\mathrm{(1)}}$ are the turnover number and the Michaelis-Menten constant of the first enzyme, respectively; ${k}_{\ell D}=4\pi \ell D$ is the rate due to diffusion over distance $\ell$ (measured in units on nm^3^/s or, after the appropriate conversion, in units of nM^−1^s^−1^), where *D* is the mutual diffusion coefficient of the first enzyme and intermediates (GOx and hydrogen peroxide for the GOx-HRP system). Figure 1b shows a reasonably good agreement between equation (1) and full numerical calculations.

Physical interpretation of equation (1) is simple. Neglecting the $\ell /b$ term for simplicity, we found that channeling increased reaction rate when ${k}_{{{\rm{E}}}_{1}}\gg {k}_{\ell D}{[{\rm{I}}]}_{{\rm{bulk}}}$. Under these conditions, the local production of intermediates would be much faster than the rate at which they diffuse away. Clearly, in this case the concentration of intermediates would be enhanced locally at the first enzyme, and hence the reaction velocity could benefit from the enzyme proximity.

Thus, proximity could enhance reaction velocity, but only when the concentration of intermediates in a solution is low, which occurs during the initial stage of reaction, as discussed, or in the presence of competing enzymes. We analysed the effect of competing enzymes by introducing the degradation rate of intermediates *k*_deg_ (see Methods); the consumption by competing enzymes was taken into account by setting ${k}_{\deg }\approx [{{\rm{E}}}_{{\rm{co}}}]{k}_{{\rm{cat}}}^{{\rm{co}}}/{K}_{M}^{{\rm{co}}}$ for low concentrations of intermediates, $[{\rm{I}}]\ll {K}_{M}^{{\rm{co}}}$, where ${K}_{M}^{{\rm{co}}}$ and ${k}_{{\rm{cat}}}^{{\rm{co}}}$ are the Michaelis-Menten constant and the turnover number, respectively, and [E_co_] the concentration of a competing enzyme. The reaction velocities of the channeled and non-channeled GOx-HRP systems, as functions of the concentration of catalase (a competing enzyme that decomposes H_2_O_2_ into water and oxygen), showed that the reaction could be accelerated by proximity channeling, but only at high catalase concentrations, with the threshold concentration increasing for increasing concentration of GOx-HRP complexes (Fig. 1c). We compiled our data on a channeling diagram (Fig. 1d), which showed the regimes under which channeling does or does not impact the reaction velocity.

In order to estimate the effect of channeling in the presence of competing enzymes, we replaced the bulk concentration of intermediates in equation (1), [I]_bulk_, by the steady-state value for a system with non-zero *k*_deg_. This turned out to provide a good approximation for the actual reaction velocity (see below) because the effect of degradation or competing consumption was negligible on the length scales determined by the separation of enzymes in an enzyme-enzyme conjugate. Indeed, in our case the main contribution to enhancement was due to the difference between the bulk and local (at the second enzyme) concentration of intermediates. Straightforward calculations gave a simple expression for the *degree of acceleration* (Supplementary Note S4B)

2 $$\delta v=({v}_{{\rm{ch}}}-{v}_{{\rm{non}}})/{v}_{{\rm{non}}}\approx \frac{{k}_{{\rm{\deg }}}+{k}_{{{\rm{E}}}_{2}}[{\rm{E}}]}{{k}_{\ell D}[{\rm{E}}]}(1-\ell /b),$$

where [E] is the concentration of enzyme complexes ([E] ≡ [E_12_] = [E_1_] = [E_2_]) and ${k}_{{{\rm{E}}}_{2}}={k}_{{\rm{cat}}}^{(2)}/{K}_{M}^{(2)}$ is the rate constant of the second enzyme (in units of nM^−1^s^−1^), assuming $[{\rm{I}}]\ll {K}_{M}^{\mathrm{(2)}}$.

Equation (2) has two limiting cases. For ${k}_{{\rm{\deg }}}\gg {k}_{{{\rm{E}}}_{2}}[{\rm{E}}]$ the proximity-induced rate enhancement is influenced by how slowly the intermediates diffuse on the time scale set by the degradation rate. In this case, straightforward algebra gave a (*δv* + 1)-fold faster rate due to channeling for the concentrations of enzyme complexes $[{\rm{E}}] < {k}_{{\rm{\deg }}}/({k}_{\ell D}\delta v)$.

In the opposite case, ${k}_{{{\rm{E}}}_{2}}[{\rm{E}}]\gg {k}_{{\rm{\deg }}}$, we have $\delta v\approx {k}_{{{\rm{E}}}_{2}}/{k}_{\ell D}$, and the reaction acceleration is determined by how slowly the intermediates diffuse on the time scale set by the reaction rate of the second enzyme. In this diffusion-limited case, fast conversion of intermediates by the second enzyme implies their (locally) low concentration, making it beneficial to keep the two enzymes together in order to increase [I] locally at the second enzyme. Under these conditions, the effect of channeling was independent of the enzyme concentration and degradation rate (but the concentration of intermediates away from an enzyme complex was kept the same as in the non-channeled system).

In a general case, but neglecting the $\ell /b$ term for simplicity (which is valid for low $[{\rm{E}}]\ll \mathrm{3/(4}\pi {\ell }^{3})\approx 0.4$ mM for $\ell =10$ nm), we obtained a simple linear relation between the concentration of enzyme complexes and the degradation rate of intermediates

3 $${[{\rm{E}}]}_{{\rm{thr}}}=\frac{{k}_{{\rm{\deg }}}}{{k}_{\ell D}\delta v-{k}_{{{\rm{E}}}_{2}}}.$$

This equation gives the concentration of enzyme complexes at which enzyme proximity provides acceleration *δv* at a given rate of degradation or competing consumption of intermediates. Note that [E]_thr_ is inversely proportional to *δv*, hence a higher *δv* will be obtained in a system with the concentration of enzymes below [E]_thr_. In other words, [E]_thr_ can be viewed as a threshold value *below* which the proximity of enzymes leads to an acceleration higher than *δv*. This is shown in Fig. 1d, where equation (3) is represented by the solid line, showing an excellent agreement with the full numerical calculations. Thus, equations (2) and (3) can be convenient estimators of proximity-induced rate enhancement.

Taking now the values of the rate constants from Zhang *et al*.^34^, and using ${k}_{{\rm{\deg }}}=[{\rm{CAT}}]{k}_{{\rm{cat}}}^{{\rm{CAT}}}/{K}_{M}^{{\rm{CAT}}}\approx$ $6.35\times {10}^{-3}[{\rm{CAT}}]$, we found ${[{{\rm{GO}}}_{{\rm{x}}}-\mathrm{HRP}]}_{{\rm{thr}}}\approx 2.8\times {10}^{-5}[{\rm{CAT}}]$ for *δv* = 2 and $\ell =8.9$ nm. For [GOx-HRP] =0.77 nM of ref.^34^, we found the *threshold* concentration, *above* which proximity channeling could give a noticeable (more than two-fold) increase in reaction velocity, [CAT]_thr _≈ 28 *μ*M. The catalase concentrations in all cases studied in ref.^34^ were a few *orders* of magnitude lower than [CAT]_thr_ (see Fig. 5 of ref.^34^ the highest value from this reference is marked by the diamond in Fig. 1d).

In order to estimate the values of *k*_cat_/*K*_*M*_ for which channelling could accelerate the overall reaction rate, we took the diffusion constant of substrates *D* = 0.6 nm^2^/ns, corresponding to glucose, and the distance between the active sites of two enzymes $\ell =1$ nm. Using equation (2), we found that enzyme proximity could enhance reaction velocity if at least one of the rate constants, either of the second enzyme of a cascade, ${k}_{{{\rm{E}}}_{2}}={k}_{{\rm{cat}}}^{(2)}/{K}_{M}^{\mathrm{(2)}}$, or of a competing enzyme, ${k}_{{\rm{cat}}}^{{\rm{co}}}/{K}_{{\rm{M}}}^{{\rm{co}}}$, would be comparable to or greater than ≈5 nM^−1^ s^−1^. While there are enzymes able to catalyze reactions at rates ∼10^9^ M^−1^ s^−1^ (*e.g.* triosephosphate isomerase and carbonic anhydrase), such high rates are rather rare in enzyme kinetics^37^. Thus, an ‘average’ cascade is unlikely to benefit from enzyme proximity in terms of its reaction velocity. However, our estimate has been done for comparable enzyme concentrations ([E] ≈ [E_co_]), increasing the concentration of competing enzymes, [E_co_], can make proximity channeling beneficial also for slower enzymes.

**Figure 2:**
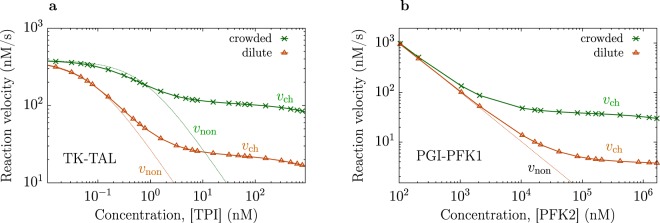
Effect of proximity channeling in dilute and crowded systems. (**a**) Reaction velocities for channeling of glyceraldehyde 3-phosphate (g3p, intermediate) in the transketolase-transaldolase (TK-TAL) part of the pentose phosphate pathway. A competing enzyme was triose-phosphate isomerase (TPI), which is diffusion limited. (**b**) Reaction velocities for channeling of fructose 6-phosphate (f6p, intermediate) in the glucose-6-phosphat-isomerase and phosphofructokinase-1 (PGI-PFK1) part of the glycolysis. A competing enzyme was phosphofructokinase-2 (PFK2). All enzymes in (**b**) are activity limited. The diffusion coefficients of g3p and f6p were taken *D* = 0.6 nm^2^/ns in a dilute and *D* = 0.06 nm^2^/ns in a crowded system. Dash lines show the reaction velocities for the non-channeled systems. The concentration of enzyme and enzyme conjugates were 26 nM in all systems.

Equation (2) shows that the effect of enzyme proximity becomes more pronounced when the diffusion of intermediates slows down. Indeed, in this case ${k}_{\ell D}$ decreases, leading to a higher value of *v*_ch_/*v*_non_. Reduced rates of diffusion occur under crowding conditions *in vivo*, where the diffusion coefficients are an order of magnitude lower than in a diluted (typical *in vitro*) system^38–44^. Figure 2a shows the effect of crowding on reaction velocities for the transketolase-transaldolase (TK-TAL) system of the pentose phosphate pathway, with glyceraldehyde 3-phosphate (g3p) as an intermediate (Supplementary Note S2). In order to demonstrate this effect, we selected triose-phosphate isomerase (TPI) as a competing enzyme. We found that, perhaps counter-intuitively, crowding increased the overall reaction rate (all considered reactions, except of TPI-catalyzed, were activity-limited, and we assumed their rates crowding-independent; for many enzymes, dependence on crowding is indeed weak^45,46^, as compared to the effect of channeling). In addition, the regime where channeling enhanced the reaction velocity comparing to the non-channeled system was shifted to higher TPI concentrations. The reason is that TPI is diffusion limited and has a lower efficiency in the crowded system, hence a higher concentration of TPI was required to induce a comparable effect.

Figure 2b shows the effect of crowding on the glucose-6-phosphate isomerase (PGI) and phosphofructokinase-1 (PFK1) system of the glycolytic pathway (Supplementary Note S3), with PFK2 as a ‘competing enzyme’ and fructose-6-phosphate as an intermediate. In this case all enzymes were activity-limited; here, crowding shifted the region where channeling became beneficial to lower PFK2 concentrations. This is because ${k}_{\ell D}$ decreased, while ${k}_{{\rm{cat}}}^{{\rm{co}}}/{K}_{{\rm{M}}}^{{\rm{co}}}$ (and hence *k*_deg_, see equation (2)) remained the same, as the system became crowded.

To summarize, we have examined the behaviour of cascade reactions when enzymes are in a close proximity to each other, which is known as proximity channeling. We have shown that the reaction velocity of the popular GOx-HRP cascade is practically independent of channeling for concentrations under typical operating conditions (Fig. 1b–d), thus rationalizing *quantitatively* the recent experimental results of Zhang *et al*.^34^. We also showed that, in general, channeling may lead to orders of magnitude increases in reaction velocities, but a significant enhancement can be obtained only for diffusion-limited reactions (with ${k}_{{\rm{cat}}}/{K}_{M}\mathop{ > }\limits_{ \tilde {}}{10}^{9}$ M^−1^ s^−1^) or at high concentrations of competing enzymes. We provide easy-to-use formulae, equations (2, 3), which allow one to estimate the effect of channeling in biotechnological applications and under *in vivo* conditions prior to experiments. These equations corroborate that channeling may have a more pronounced effect *in vivo*, where diffusion of enzymes and metabolites is significantly slowed down^38–44^. In particular, crowding enhances the overall rates of channeled cascade reactions, and can shift the region where proximity becomes more effective towards higher or lower concentrations of competing enzymes, depending on enzyme efficiencies (Fig. 2). Thus, while it is interesting to analyse proximity channeling with *in vitro* systems, it is vital to consider the effect *in vivo* in order to reveal the biological and evolutionary role, and the physiological significance of formation of enzyme complexes in living cells^15,21,47^.

## Methods

The intermediate substrates are described by a continuous field *C*(*r*, *t*) ≡ [I] that satisfies the diffusion equation

4 $$\frac{dC}{dt}=D{\nabla }^{2}C-{k}_{\deg }C,$$

where *k*_deg_ is the degradation rate and/or the rate due to competing side reactions, and *D* is the mutual diffusion coefficient of intermediates and enzyme-enzyme complexes.

The enzymes are modeled as spherical particles containing small active sites with opening angle *α* on their surfaces (Fig. 1a and Supplementary Fig. S1). The boundary conditions on the enzyme active sites ${{\rm{E}}}_{i}^{{\rm{as}}}$ are

5a $$\begin{array}{cc}D\hat{{\rm{n}}}\cdot \nabla C(r,t)=-\,{k}_{1}, & r\in \partial {{\rm{E}}}_{1}^{{\rm{as}}}\end{array}$$

for the first enzyme, and

5b $$\begin{array}{cc}D\hat{{\rm{n}}}\cdot \nabla C(r,t)=-\,{k}_{2}C(r,t), & r\in \partial {{\rm{E}}}_{2}^{{\rm{as}}}\end{array}$$

for the second enzyme of a cascade, and we applied von Neumann boundary conditions ($\hat{{\rm{n}}}\cdot \nabla C=0$) to the rest of the enzyme surfaces. Here $\hat{{\rm{n}}}$ is a unit vector normal to the surface.

The total production rate is ${v}_{{\rm{ch}}}=({k}_{2}/V){\int }_{\partial {{\rm{E}}}_{2}^{{\rm{as}}}}C(r)dS$, where *V* is the volume of a computational box and *dS* the surface element. We need to solve only the time independent equation, *dC*/*dt* = 0, in order to find the steady-state reaction velocity, of interest in this work.

In steady-state the appropriate boundary condition away from the enzymes is vanishing concentration gradient (von Neumann boundary condition). In this case, and setting *k*_deg_ = 0, we obtained by integrating equation (4) once

6 $${v}_{{\rm{ch}}}=\frac{{k}_{2}}{V}{\int }_{\partial {{\rm{E}}}_{2}^{{\rm{as}}}}C(r)dS=\frac{{k}_{1}}{V}{\int }_{\partial {{\rm{E}}}_{1}^{{\rm{as}}}}dS=\frac{{k}_{1}{A}_{1}}{V}\equiv {v}_{{\rm{non}}},$$

where *A*_1_ is the surface area of the active site of the first enzyme. This equation expresses the conservation law and means that, in steady state and for *k*_deg_ = 0, the production rate is determined *solely* by the production rate of the first enzyme, which is exactly the production rate in the non-channelled system (see equation (11) and below). This argument does not apply to systems that are not in steady state or if *k*_deg_ ≠ 0.

The solution to equation (4) has been obtained numerically using F3DM library^48^. We applied von Neumann boundary condition away from the enzymes to produce all plots, except of Fig. 1a,b (where we kept the concentration of intermediates fixed, as denoted on the plots). The analytical steady-state solution for the system with just the first enzyme allowed us to obtain approximate equations (1, 2); the derivation is presented in Supplementary Note S4.

Similar approaches have been used before and proved to provide correct results^6,49^.

### Homogeneous non-channeled system

We also considered an equivalent homogeneous bulk system, where enzymes do not form complexes and are well mixed. An equation describing the evolution of this system is

7 $$\frac{d[{\rm{I}}]}{dt}=[{{\rm{E}}}_{1}]{k}_{{{\rm{E}}}_{1}}-{k}_{{\rm{\deg }}}[{\rm{I}}]-{k}_{{{\rm{E}}}_{2}}[{{\rm{E}}}_{2}][{\rm{I}}],$$

where ${k}_{{{\rm{E}}}_{i}}$, *i* = 1, 2, are enzyme’s rate constants, *k*_deg_ the degradation rate of intermediates, [I] is the concentration of intermediates and [E_*i*_] the concentration of enzyme E_*i*_. The production rate is ${v}_{{\rm{non}}}={k}_{{{\rm{E}}}_{2}}[{{\rm{E}}}_{2}][{\rm{I}}]$.

Equation (7) obeys a simple analytical solution

8 $$[{\rm{I}}](t)=[{{\rm{E}}}_{1}]{k}_{{{\rm{E}}}_{1}}\tau \mathrm{(1}-{{\rm{e}}}^{-t/\tau }),$$

where $\tau =\mathrm{1/(}{k}_{{\rm{\deg }}}+{k}_{{{\rm{E}}}_{2}}[{{\rm{E}}}_{2}])$ is the relaxation or decay constant. Incidentally, this equation gives for the time at which [I](*t*) = [I]_0_

9 $${t}_{0}=-\,\tau \,\mathrm{ln}(1-\,{[{\rm{I}}]}_{0}/{k}_{{{\rm{E}}}_{1}}[{{\rm{E}}}_{1}]\tau )\mathrm{.}$$

The steady-state concentration of intermediates is

10 $${[{\rm{I}}]}_{{\rm{SS}}}=[{\rm{I}}](t=\infty )=\frac{[{{\rm{E}}}_{1}]{k}_{{{\rm{E}}}_{1}}}{{k}_{{{\rm{E}}}_{2}}[{{\rm{E}}}_{2}]+{k}_{\deg }},$$

and hence the reaction velocity

11 $${v}_{{\rm{non}}}=\frac{{k}_{{{\rm{E}}}_{2}}[{{\rm{E}}}_{2}]{k}_{{{\rm{E}}}_{1}}[{{\rm{E}}}_{1}]}{{k}_{{{\rm{E}}}_{2}}[{{\rm{E}}}_{2}]+{k}_{{\rm{\deg }}}},$$

which becomes ${v}_{{\rm{non}}}={k}_{{{\rm{E}}}_{1}}[{\rm{E}}]$ for *k*_deg_ = 0 if we take the same enzyme concentrations [E] = [E_1_] = [E_2_] in order to compare with the channeled system.

To relate enzyme concentrations in the non-channeled system and in the channeled system of Fig. 1a, we considered the computational box as a ‘unit cell’ so that the corresponding enzyme concentration [E_*i*_] = 1/*V*, where *V* is the box volume. The rate constants *k*_*i*_ and ${k}_{{{\rm{E}}}_{i}}$ are related by ${k}_{{{\rm{E}}}_{i}}={A}_{i}{k}_{i}$, where *A*_*i*_ is the surface area of the active site of the *i*’th enzyme. These relations imply that for *k*_deg_ = 0 the steady-state velocities of the channeled and non-channeled systems are the same (equations (6) and (11)).

## Supplementary information

Reaction parameters and the derivation for the degree of acceleration
