# Supplementary material for: Can enzyme proximity accelerate cascade reactions?
Source: Sci Rep. 2019 Jan 24;9:455. doi: 10.1038/s41598-018-37034-3 (PMC6345930; doi:10.1038/s41598-018-37034-3)
Supplement: Supplementary file 1 — Reaction parameters and the derivation for the degree of acceleration [file 41598_2018_37034_MOESM1_ESM.pdf]

Can enzyme proximity accelerate cascade reactions? Supplementary Information.

Andrij Kuzmak, Sheiliza Carmali, Eric von Lieres, Alan J. Russell, and Svyatoslav Kondrat

S1. GOX-HRP CASCADE

The GOx-HRP cascade consists of glucose oxidase (GOx) and horseredish peroxidase (HRP). In our calculations we have taken the parameters consistent with [1]; the details of the calculations and the parameters are listed below.

A. Glucose oxidase

We modeled GOx as a spherical particle of radius $R_1 = 4.3$nm; the active site surface area was $A_1 = 1.0135\text{nm}^2$ (the surface area was calculated using computer program Maestro 11.6 [2]), which amounts to angle $\alpha = 7.58°$ in our model (see Fig. 1a and *cf.* Fig. S1). The activity of GOx (*i.e.* the rate of production of the hydrogen peroxide) depends on the concentration of Glucose (Glu) as follows

$$v_{\mathrm{H_2O_2}} = \frac{[\mathrm{GOx}][\mathrm{Glu}]k_{\mathrm{cat}}}{K_M + [\mathrm{Glu}]} \tag{S1}$$

where $k_{\mathrm{cat}} = 250\text{s}^{-1}$ and $K_M = 15$mM.

In our model, the rate of production of H_2O_2 was (see equation (5a) in the main text)

$$v_{\mathrm{H_2O_2}} = \frac{k_1 A_1}{V} \tag{S2}$$

where $V = 1/[\mathrm{GOx}]$ is the volume (of a computational box), and k_1 the production rate per surface area. Thus

$$k_1 = 1.67\text{nM} \cdot \text{nm}\, \frac{250\text{s}^{-1} \cdot [\mathrm{Glu}]}{15\text{mM} + [\mathrm{Glu}]}, \tag{S3}$$

where the factor 1.67 comes from units conversion. For 1mM Glucose we obtained $k_1 \approx$ 26.1nM nm/ns. We used this value in all calculations.

B. Horseradish peroxidase

Similarly to GOx, we modeled HRP as a spherical particle of radius $R_2 = 3.6$nm; the active site surface area was $A_2 = 0.3523\text{nm}^2$ (the surface area was calculated using computer program Maestro 11.6 [2]), which amounts to angle $\alpha = 5.33°$. The activity is $k_{\text{cat}}/K_M = 13.08 \times 10^{-3}\text{nM}^{-1}\text{s}^{-1}$, where $K_M = 2.5\mu$M and $k_{\text{cat}} = 32.7\text{s}^{-1}$.

In order to obtain k_2 for our model (see equation (5b) in the main text), we needed to perform separate numerical calculations. However, since the reaction is activity limited (the diffusion coefficient of hydrogen peroxide is $D = 1.8\text{nm}^2/\text{s}$), k_2 could be well approximated by

$$k_2 \approx \frac{k_{\text{cat}}/K_M}{0.6A_2}, \tag{S4}$$

where 0.6 comes from unit conversion. We obtained $k_2 \approx 0.062$nm/ns, which we used in all calculations.

C. Catalase

Catalase (CAT) is an enzyme competing with HRP for hydrogen peroxide. In our calculations the parameters were $k_{\text{cat}} = 6.1 \times 10^5\text{s}^{-1}$ and $K_M = 96$mM so that $k_{\text{cat}}/K_M \approx 6.35 \times 10^{-3}\text{nM}^{-1}\text{s}^{-1}$. We assumed small hydrogen peroxide concentrations ($[H_2O_2] < K_M$) so its rate of consumption by catalase $[\text{CAT}][H_2O_2]k_{\text{cat}}/K_M$ and $k_{\text{deg}} = [\text{CAT}]k_{\text{cat}}/K_M$.

S2. TK-TAL CASCADE

Transketolase (TK) and transaldolase (TAL) are part of non-oxidative phase of the pentose phosphate pathway. TK has two substrates, xylulose 5-phosphate and ribose 5-phosphate, and likewise two products, glyceraldehyde 3-phosphate (g3p) and sedoheptulose 7-phosphate, which are transformed by TAL to erythrose 4-phosphate and fructose 6-phosphate. We considered g3p as an intermediate that is competitively consumed by triose-phosphate isomerase (TPI).

We assumed that the enzymes are close to each other (1 nm surface-to-surface distance) but rotate freely. Thus, unlike for the GOx-HRP cascade, we modeled TK-TAL as spherical enzymes with the average activity homogeneously distributed over the enzyme surface.

A. TK

We modeled TK as a spherical enzyme of radius $R_1 = 4.43$nm and considered a constant production rate of g3p $v_{\mathrm{g3p}} = 15$ nM/s, which amounts to $k_1 = 0.1$ nM nm/ns within our model.

B. TAL

Similarly to TK, we modeled TAL as a spherical particle of radius $R_2 = 5.51$nm and took $k_{\mathrm{cat}}/K_M = 6.6 \times 10^3$ mM^{-1}s^{-1} [3]. Since the reaction is activity limited, we used equation (S4) which gave $k_2 = 0.016$nm/ns.

C. TPI

We have taken TPI as a competing enzyme. It catalyzes (reversibly) g3p to dihydroxyacetone phosphate. We neglected the reverse reaction for simplicity, and took $K_{\mathrm{TPI}} = k_{\mathrm{cat}}/K_M = 1269000000$ mM^{-1} s^{-1} [4]. Since intrinsic association/disassociation constants were not know to us, in order to take into account crowding, we assumed that TPI is catalytically perfect and scaled K_{TPI} by the ratio of the diffusion constants.

S3. PGI-PFK CASCADE

Phosphoglucose isomerase (PGI) and phosphofructokinase-1 (PFK1) are part of the Glycoltic pathway. The substrate for PGI is glucose 6-phosphate (g6p), which is transformed into fructose 6-phosphate (f6p); the f6p is then catalyzed by PFK1 into fructose 1,6-bisphosphate (f1,6p). We assumed that the enzymes are close to each other and rotate freely. We modeled PGI and PFK as spherical enzymes with the average activity homogeneously distributed over the enzyme surface.

A. PGI

We modeled PGI as a spherical enzyme of radius $R_1 = 5.64$nm with the parameters $k_{\text{cat}} = 10^3\text{s}^{-1}$ and $K_M = 0.31$mM. Since PGI catalyzes both forward and backward reactions, we took 70% of the velocity of the forward reaction to account for the backward reaction [5]. Then applying the same reasoning as for GOx, we obtained for k_1 (see equation (5a))

$$k_1 = \frac{0.7k_{\text{cat}}}{A_1}\frac{[\text{g6p}]}{K_M + [\text{g6p}]} = \frac{2.92\text{nM} \cdot (\text{nm/ns}) \cdot [\text{g6p}]}{0.31\text{mM} + [\text{g6p}]}, \tag{S5}$$

where A_1 is the surface area of PGI. For a typical intracellular concentration [g6p] = 40mM we get $k_1 = 3.83$nM nm/ns.

B. PFK1

Similarly to PGI, we modeled PFK1 as a spherical particle of radius $R_2 = 3.24$nm and took $k_{\text{cat}} = 88\text{s}^{-1}$ and $K_M = 0.011$mM. Since the reaction is activity limited, we used equation (S4) which gives $k_2 = 10^{-4}$nm/ns.

C. PFK2

We have taken PFK2 as a 'competing enzyme'. It takes fructose-6-phosphate (PGI-PFK1 intermediate) to produce fructose-2,6-phosphate. The parameters were $k_{\text{cat}} = 53\text{s}^{-1}$ and $K_M = 0.1$mN, which gave $k_{\text{deg}} = [\text{PFK2}]k_{\text{cat}}/K_M = 0.53 \times 10^{-3}[\text{PFK2}]$ in crowded and dilute systems.

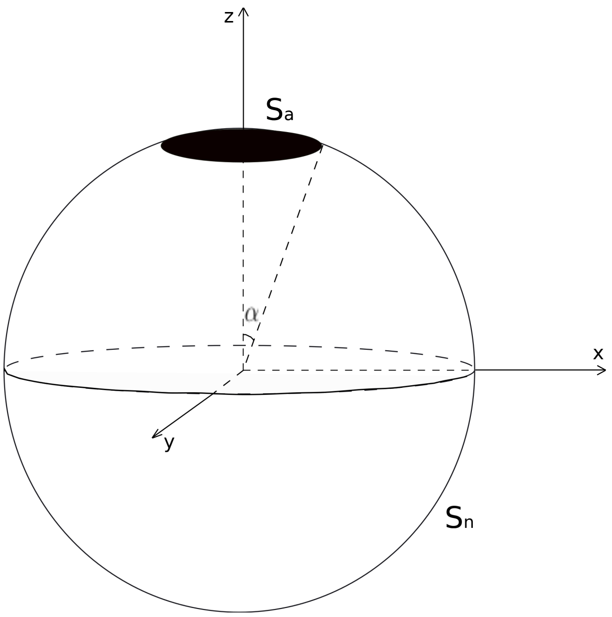

FIG. S1. **Model of enzyme.** S_a (black shading) denotes the active size and α is half opening angle of the active site. S_n denotes the rest of the enzyme surface.

S4. APPROXIMATE EQUATIONS FOR THE EFFECT OF CHANNELING

Herein, we derive approximate equations (1) and (2), which allow one to estimate the effect of enzyme proximity on reaction velocity. To this end, we considered only the first enzyme of a cascade, which produces intermediates, and we calculated the concentration of intermediates, C, at position $\boldsymbol{r}_2$ of the active site of the second enzyme. The reaction velocity is then

$$v_{\mathrm{ch}} = k_{\mathrm{E}_2}[\mathrm{E}_2]C(\boldsymbol{r}_2), \tag{S6}$$

where k_{E_2} is the reaction rate of the second enzyme and $[\mathrm{E}_2]$ is its concentration.

The concentration of intermediates satisfies the stationary diffusion equation

$$\nabla^2 C = 0, \tag{S7}$$

with the following boundary conditions on the enzyme surface

$$\mathbf{n}\cdot\nabla C\bigg|_{S_n} = 0, \quad D\mathbf{n}\cdot\nabla C\bigg|_{S_a} = kg\bigg|_{S_a}, \tag{S8}$$

where D is the mutual diffusion coefficient of intermediates and the enzyme, k is the rate constant (see equation (5a)), g is a function to be specified below, $\mathbf{n}$ is a unit vector, S_a is

the active site and S_n the remaining enzyme surface (Fig. S1). To solve this problem we direct the z-axis through the center of the active site and use spherical coordinate system; we have

$$\frac{1}{r}\frac{\partial^2}{\partial r^2} rC + \frac{1}{r^2}\frac{1}{\sin\theta}\frac{\partial}{\partial\theta}\left(\sin\theta\frac{\partial}{\partial\theta}\right)C = 0, \tag{S9}$$

and

$$D\frac{\partial C}{\partial r}\bigg|_{r=a} = kg\bigg|_{r=a} H(\alpha-\theta), \tag{S10}$$

where r is the radial distance and θ the polar angle, a is the enzyme radius, α is the half the opening angle of the active site and $H(\alpha-\theta)$ is the Heaviside step function, which is unity for $\theta < \alpha$ and it is zero otherwise. Finally, we assumed a constant (bulk) concentration far from the enzyme, *i.e.*,

$$C\bigg|_{r=b} = C_{\text{bulk}}. \tag{S11}$$

After separation of variables we obtained

$$\frac{1}{\sin\theta}\frac{\partial}{\partial\theta}\left(\sin\theta\frac{\partial\Theta}{\partial\theta}\right) + \lambda\Theta = 0, \tag{S12}$$

$$r^2\frac{\partial^2 R}{\partial r^2} - \lambda R = 0, \tag{S13}$$

where $C = f\Theta$ and $R = rf$. Here f and Θ depend on the radial distance and polar angle, respectively. The solution of the first equation is a Legendre Polynomials $P_n(\cos\theta)$ with $\lambda_n = n(n+1)$, where $n = 0, 1, \ldots$. The solution of the second equation takes the form

$$R_n = A_n r^{n+1} + B_n r^{-n}. \tag{S14}$$

Then the general solution of equation (S9) is

$$C = \sum_{n=0}^{\infty}\left(A_n r^n + B_n r^{-(n+1)}\right)P_n(\cos\theta). \tag{S15}$$

We then took $g = -1$ in equation (S10), which corresponds to the boundary condition (5a) of the main text. We obtained the system of equations for A_n and B_n

$$\begin{aligned} &A_n b^n + B_n b^{-(n+1)} = C_{\text{bulk}}\delta_{0n}, \\ &nA_n a^{n-1} - (n+1)B_n a^{-(n+2)} = -\frac{2n+1}{2}\frac{k}{D}\int_0^{\alpha} P_n(\cos\theta)\sin\theta d\theta. \end{aligned} \tag{S16}$$

Limiting our consideration to the first order in $1/r$, we solved the system (S16) and obtained for the concentration of intermediates

$$C(r) = C_{\mathrm{bulk}} + \frac{k}{D} a^2 \left(\frac{1}{r} - \frac{1}{b}\right) \sin^2 \frac{\alpha}{2}. \tag{S17}$$

Taking $r = \ell$, where ℓ is the distance to the active site of the first enzyme, and using equation (S6), we obtained for the reaction velocity

$$v_{\mathrm{ch}} = v_{\mathrm{non}} \left[1 + \frac{k_{\mathrm{E}_1}}{k_D C_{\mathrm{bulk}}} \left(\frac{a}{\ell} - \frac{a}{b}\right)\right], \tag{S18}$$

where $v_{\mathrm{non}} = k_{E_2}[E_2]C_{\mathrm{bulk}}$ is the reaction velocity of the non-channeled reaction, $k_D = 4\pi Da$ is the Smoluchowski rate due to diffusion and $k_{\mathrm{E}_1} = 4\pi k a^2 \sin^2(\alpha/2)$ is the rate constant of the first enzyme. Dividing equation (S18) by v_{non} we obtained equation (1) of the main text (where we used $k_{\ell D} = 4\pi D\ell$ in lieu of k_D for convenience).

A. Multiple active sites

Equation (S18) is valid also in the case when enzymes have more than one active site. Indeed, in this case we have for N active sites

$$D\mathbf{n} \cdot \nabla C \bigg|_{S_{ai}} = -k_i, \tag{S19}$$

where S_{ai} and k_i are the area and rate constant of ith active site, respectively. Then the solution of the stationary diffusion equation (S7) for such an enzyme up to the first order in $1/r$

$$C(r) = C_{\mathrm{bulk}} + \frac{1}{D} a^2 \left(\frac{1}{r} - \frac{1}{b}\right) \sum_{i=1}^{N} k_i \sin^2 \frac{\alpha_i}{2}, \tag{S20}$$

where α_i is half of the opening angle of the ith active site. Clearly, this equation leads to equation (S18) with $k_{\mathrm{E}} = 4\pi a^2 \sum_{i=1}^{N} k_i \sin^2(\alpha_i/2)$.

B. Degrading intermediates and competing enzymes

We also solved analytically the problem with degrading intermediates, *i.e.*, with $k_{\mathrm{deg}} \neq 0$ in equation (4) of the main text. However, the equation analogous to equation (S18) turned out to be lengthy and not physically appealing. In order to estimate the effect of channeling

in this case, we took the steady-state concentration of intermediates in the system in which intermediates do degrade, and used it in equation (S18). As explained in the main text, this is possible to do as long as the effect of degradation or competing consumption is negligible on the length scales determined by the separation of enzymes in an enzyme complex.

The bulk concentration of intermediates in the system with degrading intermediates can be obtained using the homogeneous system (see equation (8) in the main text, where it is denoted by [I]; the limit $t \to \infty$ must be taken to obtain the steady-state concentration of intermediates). We obtained

$$C_{\mathrm{bulk}} = \frac{[\mathrm{E}]k_{\mathrm{E}_1}}{k_{\mathrm{deg}} + k_{\mathrm{E}_2}[\mathrm{E}]}, \tag{S21}$$

where [E] is the enzyme concentration assumed the same for both enzymes and for enzyme complexes. Using now this value of C_{bulk} in equation (S18) we obtained equation (2) of the main text.

C. Collins-Kimball equation

Incidentally, we note that the well-known Collins-Kimball equation for diffusion-controlled reactions can be obtained using the same level of approximations. We took $g = C$ in boundary condition (S10), where now C is the substrate concentration for the enzyme, and we were interested in the dependence of a *macroscopic* reaction rate on diffusion. We set $b = \infty$ to obtain the following system of algebraic equations for B_n

$$B_m = -\frac{2m+1}{2(m+1)}\frac{ka^{m+2}}{D}\left(C_{\mathrm{bulk}}J_m + \sum_{n=0}^{\infty}\frac{B_n}{a^{n+1}}J_{nm}\right), \tag{S22}$$

where

$$J_m = \int_0^{\alpha} P_m(\cos\theta)\sin\theta d\theta, \quad J_{nm} = \int_0^{\alpha} P_n(\cos\theta)P_m(\cos\theta)\sin\theta d\theta. \tag{S23}$$

The concentration up to the first order in $1/r$ reads

$$C(r) = C_{\mathrm{bulk}}\left(1 - \frac{k\sin^2\frac{\alpha}{2}}{D + ka\sin^2\frac{\alpha}{2}}\frac{a^2}{r}\right). \tag{S24}$$

The macroscopic rate constant is

$$K = \frac{1}{C_{\mathrm{bulk}}}\int_{S_a} kCdS, \tag{S25}$$

which gives upon substituting equation (S24) into equation (S25)

$$K = 4\pi \frac{Dka^2 \sin^2 \frac{\alpha}{2}}{D + ka \sin^2 \frac{\alpha}{2}} = \frac{k_D k_{\mathrm{E}}}{k_D + k_{\mathrm{E}}}, \tag{S26}$$

where $k_D = 4\pi Da$ and $k_{\mathrm{E}} = 4\pi ka^2 \sin^2(\alpha/2)$ as before. Equation (S26) is the well known Collins-Kimball equation for diffusion-controlled reactions [6, 7].

---

[1] Y. Zhang, S. Tsitkov, and H. Hess, "Proximity does not contribute to activity enhancement in the glucose oxidase-horseradish peroxidase cascade," Nature Comm. **7**, 13982 (2016).

[2] "Schrödinger Release 2018-2: Maestro," LLC, New York, NY (2018).

[3] M. Rale, S. Schneider, G. A. Sprenger, A. K. Samland, and W.-D. Fessner, "Broadening deoxysugar glycodiversity: Natural and engineered transaldolases unlock a complementary substrate space," Chemistry - A European Journal **17**, 2623–2632 (2011).

[4] P. Sharma and P. Guptasarma, "'super-perfect' enzymes: Structural stabilities and activities of recombinant triose phosphate isomerases from pyrococcus furiosus and thermococcus onnurineus produced in escherichia coli," Biochem. Biophys. Res. Commun. **460**, 753–758 (2015).

[5] A. Flamholz, E. Noor, A. Bar-Even, W. Liebermeister, and R. Milo, "Glycolytic strategy as a tradeoff between energy yield and protein cost," Proceedings of the National Academy of Sciences **110**, 10039–10044 (2013).

[6] F. C. Collins and G. E. Kimball, "Diffusion-controlled reaction rates," J. Colloid. Sci. **4**, 425–437 (1949).

[7] O. G. Berg, "On diffusion-controlled dissociation," Chemical Physics **31**, 47–57 (1978).
